# Exploring the symbiotic pangenome of the nitrogen-fixing bacterium *Sinorhizobium meliloti*

**DOI:** 10.1186/1471-2164-12-235

**Published:** 2011-05-12

**Authors:** Marco Galardini, Alessio Mengoni, Matteo Brilli, Francesco Pini, Antonella Fioravanti, Susan Lucas, Alla Lapidus, Jan-Fang Cheng, Lynne Goodwin, Samuel Pitluck, Miriam Land, Loren Hauser, Tanja Woyke, Natalia Mikhailova, Natalia Ivanova, Hajnalka Daligault, David Bruce, Chris Detter, Roxanne Tapia, Cliff Han, Hazuki Teshima, Stefano Mocali, Marco Bazzicalupo, Emanuele G Biondi

**Affiliations:** 1 Department of Evolutionary Biology, University of Firenze, via Romana 17, I-50125 Firenze, Italy; 2 Laboratoire de Biométrie et Biologie Evolutive, UMR CNRS 5558, Université Lyon 1, 43, bvd du 11 novembre, Lyon, France; 3 DOE Joint Genome Institute, Walnut Creek, California, USA; 4 Fox Chase Cancer Center, 333 Cottman Avenue, Philadelphia, USA; 5 Los Alamos National Laboratory, 1619 Central Avenue, Los Alamos, USA; 6 Oak Ridge National Laboratory, Oak Ridge, USA; 7 Agricultural Research Council- Agrobiology and Pedology Centre (ABP) P.za D'Azeglio, 30, 50121 - Firenze, Italy; 8 Interdisciplinary Research Institute - CNRS, Villenenuve d'Ascq, France

**Keywords:** Sinorhizobium meliloti, nodulation, symbiosis, comparative genomics, pangenome, panregulon

## Abstract

**Background:**

*Sinorhizobium meliloti *is a model system for the studies of symbiotic nitrogen fixation. An extensive polymorphism at the genetic and phenotypic level is present in natural populations of this species, especially in relation with symbiotic promotion of plant growth. AK83 and BL225C are two nodule-isolated strains with diverse symbiotic phenotypes; BL225C is more efficient in promoting growth of the *Medicago sativa *plants than strain AK83. In order to investigate the genetic determinants of the phenotypic diversification of *S. meliloti *strains AK83 and BL225C, we sequenced the complete genomes for these two strains.

**Results:**

With sizes of 7.14 Mbp and 6.97 Mbp, respectively, the genomes of AK83 and BL225C are larger than the laboratory strain Rm1021. The core genome of Rm1021, AK83, BL225C strains included 5124 orthologous groups, while the accessory genome was composed by 2700 orthologous groups. While Rm1021 and BL225C have only three replicons (Chromosome, pSymA and pSymB), AK83 has also two plasmids, 260 and 70 Kbp long. We found 65 interesting orthologous groups of genes that were present only in the accessory genome, consequently responsible for phenotypic diversity and putatively involved in plant-bacterium interaction. Notably, the symbiosis inefficient AK83 lacked several genes required for microaerophilic growth inside nodules, while several genes for accessory functions related to competition, plant invasion and bacteroid tropism were identified only in AK83 and BL225C strains. Presence and extent of polymorphism in regulons of transcription factors involved in symbiotic interaction were also analyzed. Our results indicate that regulons are flexible, with a large number of accessory genes, suggesting that regulons polymorphism could also be a key determinant in the variability of symbiotic performances among the analyzed strains.

**Conclusions:**

In conclusions, the extended comparative genomics approach revealed a variable subset of genes and regulons that may contribute to the symbiotic diversity.

## Background

*Sinorhizobium *(syn. *Ensifer*) *meliloti *belongs to the *Rhizobiales *order of the *alpha-Proteobacteria *class, together with important human pathogens such as *Bartonella *and *Brucella*, and with several plant-associated bacteria of relevant agricultural importance, such as *Agrobacterium, Ochrobactrum, Bradyrhizobium, Mesorhizobium *and *Rhizobium *[1]. *S. meliloti *is distributed world-wide in many soil types where it can be found in free living form or as a symbiont of leguminous (*Fabaceae*) plants, on which it induces the formation of nodules, specialized organs where bacteria fix nitrogen within the plant cytoplasm [2]. *Medicago sativa *L. (alfalfa) and the diploid relative *M. truncatula *Gaertn. (barrel medic) are among the most studied host species for the *S. meliloti *symbiosis [2-4]. Although several essential features of the symbiotic association between alfalfa (and barrel medic) and *S. meliloti *have been elucidated and, nowadays, scientists are able to explain most of the major steps of nodule formation, many aspects are still not fully understood [2]. In fact, although the main steps and genes related to symbiosis have been identified by mutants produced in laboratory (see NodMutDB, [5]), one of the less considered aspects of the rhizobium-legume symbiosis concerns the effects of genetic variation of natural strains on plant growth due to differences in symbiotic efficiency. In this perspective, two of the most investigated strains of *S. meliloti *are BL225C and AK83 [6]. These strains were isolated while investigating the genetic variability of *S. meliloti *populations ([7] and M. Roumiantseva, unpublished results) and revealed different symbiotic phenotypes [8]. Strain BL225C was found to be more effective in increasing plant growth of *M. truncatula *and alfalfa plants than strain AK83; indeed plants inoculated with AK83 grow similar to un-inoculated control plants even though they produce a larger number of immature nodules. Comparative genomic hybridization (CGH) studies showed that AK83 and BL225C strains have from 5.7% to 6.5% of CDS divergent (mutated or deleted) with respect to the reference sequenced strain Rm1021 [6], most of the genomic polymorphism being located on the symbiotic megaplasmid pSymA. However, a CGH array can only reveal when genes present in the microarray, represented by the reference genome, are lost or duplicated in the other strains, but it is unable to identify the genetic repertoire exclusively possessed by a novel strain. To date, the only genome sequence available for *S. meliloti *belonged to strain Rm1021 [9] and only recently also to strain SM11 [10]. However it is known that most of the genomic analyses in bacteria revealed large differences in genes content even between closely related strains (for a review see [11]) justifying the introduction of the pangenome concept [12,13] where the pangenome is intended as the sum of "core" (conserved in all strains) and "accessory" (variable among strains) genes. It has also been proposed that non-essential genes are responsible for driving the evolutionary diversification between bacterial strains [14], even if their adaptive value is often uncertain [15]. Despite the large interest and the number of studies performed on *S. meliloti *biology and genetics, the size and the functionalities of the *S. meliloti *pangenome remain to be extensively elucidated, especially at the level of the symbiotic diversity.

Moreover, besides the gene content present in the accessory genome, also regulatory networks have been shown to be plastic enough to accommodate and explain phenotypic variability at different evolutionary scales [15-17]. In bacteria, regulon polymorphism, that is the existence of a core and an accessory regulon, has been previously studied in different contexts and taxonomic scopes, such as pathogenesis regulation in *Clostridium perfringens *strains [18] and cell cycle control in the alpha-proteobacteria class [19], both at the intra-specific and inter-specific levels, respectively. In particular, it was shown that in some alpha-proteobacteria the cell-cycle regulatory circuits undergo rearrangements which seem to maintain the logic of the regulation.

In this work, we present the genome sequences of *S. meliloti *strains AK83 and BL225C, aiming to provide a depiction of the *S. meliloti *pangenome, which may be associated with symbiotic interaction and then could be at the basis of differences in the symbiotic efficiency of natural strains. To address this aim, after full genome sequencing and annotation, we developed a pipeline of automatic search which integrates available general purpose genomic databases (NCBI, KEGG, InterPro) with rhizobial specific resources (Rhizobase Bibliome and the nodMutDB [5]) to identify all genes that could be possibly related to symbiosis. Then we investigated the possible genetic determinants of phenotypic differences between these strains using computational methods and integrating classical genomics analysis, such as the identification of shared and specific genes with the prediction of regulons for selected transcription factors that are known to play a role during symbiosis. Together, these approaches allowed us to find a genomic interpretation to the phenotypic differences and to define a set of accessory genetic factors related to the symbiotic process.

## Results and Discussion

### General features of AK83 and BL225C genomes

The genome sequences of strains AK83 and BL225C, were obtained as described in Materials and Methods; both genomes resulted to be larger than that of strain Rm1021 by additional 450 kbp (AK83) and 290 kbp (BL225C) (Table 1). This larger DNA content is paralleled by an increase in the number of CDSs (from 6218 of Rm1021 to 6518 and 6359 of the mentioned sequenced strains, respectively).

**Table 1:** General genomic features of AK83 and BL225C strains in comparison with Rm1021

	Rm1021*	AK83	BL225C
Length (Mb)	6.69	7.14	6.98
G+C content	61.3%	61.9%	62.0%
Coding	86.1%	85.6%	84.7%
ORFs	6218	6518	6359
rRNA	9	9	9
tRNA	54	56	55
Chromosome (Mb)	3.65	3.82	3.67
Chromid pSymB (Mb)	1.68	1.68	1.69
Megaplasmid pSymA (Mb)	1.35	1.31	1.61
Plasmid 1 (Mb)	NP	0.26	NP
Plasmid 2 (Mb)	NP	0.07	NP
ORFs with no function	23.72%	28.86%	24.60%
ORFs with no similarity (ORFan)	3.22%	5.63%	3.74%
ORFs annotated by COG	76.28%	71.14%	75.40%
ORFs annotated by Interpro	85.70%	82.60%	84.29%
ORFs annotated by GO	66.13%	62.44%	64.16%
ORFs annotated by KEGG	55.53%	54.88%	54.32%
ORFs with homology with members of Rhizobase**	92.46%	87.63%	90.64%
Putative transposases	152	135	76
Putative Type III secretion systems-related proteins	5	5	5
Putative Type IV secretion systems-related proteins	4	8	7
Putative Two component systems-related proteins	129	126	134
Putative ABC transporters-related proteins	314	302	309

* Data from NCBI genome database http://www.ncbi.nlm.nih.gov/sites/entrez?db=genome

** Not considering strain Rm1021

NP: not present

Strain AK83 has the highest percentage of ORFans (orphan ORFs) (5.63%), defined as those genes with no detectable similarity with other genes in any other organism [20], and the lowest number of ORFs coding for proteins with homology to COGs, InterPro, GO and Rhizobase entries, while strain Rm1021 shows the highest number of transposases and Insertion Sequences (152) compared to AK83 and BL225C (135 and 76, respectively). Other relevant features of general importance are those related to the environmental sensing and transport: we noticed that BL225C and AK83 strains have more Type IV secretion systems-related proteins than the reference strain Rm1021. However, a similar number of ABC transporters-related proteins and of Type III secretion systems-related proteins were found across the three genomes, tough no fully functional Type III secretion systems were detected, as previously noticed for Rm1021 strain [9]. Finally, two-component signal transduction systems related proteins are slightly higher in Rm1021 and BL225C strains than in AK83 (129, 134 and 125, respectively).

### Defining core and accessory *S. meliloti *genome

**Figure 1:**
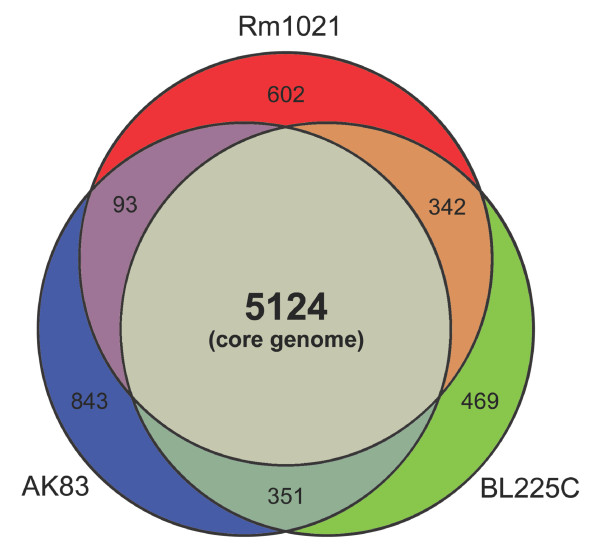
**Size of core and accessory genome of three *S. meliloti *strains**. The number of orthologous groups found in each intersection is reported. Areas are not in scale.

By comparing the 19095 CDSs, found in the three genomes, a set of 7824 orthologous groups was identified; a subset of 5124 was conserved across all the three genomes and accordingly defined as the core genome of *S. meliloti *species. The remaining 2700 orthologous groups were defined as members of the accessory genome for these three genomes. The strain with more unique genes is AK83, with 843 exclusive groups, while BL225C and Rm1021 have 469 and 602 exclusive groups, respectively (Figure 1). In Additional file 1 the full list of core and accessory proteins is reported.

When the very recently published SM11 genome [10] was added to our proteome set of the three strains (AK83, BL225C, Rm1021), the core genome contained 5075 orthologous groups, with the loss of 49 groups, suggesting a certain stability of the core genome size in this species, while the accessory genome comprised 3810 orthologous groups.

**Figure 2:**
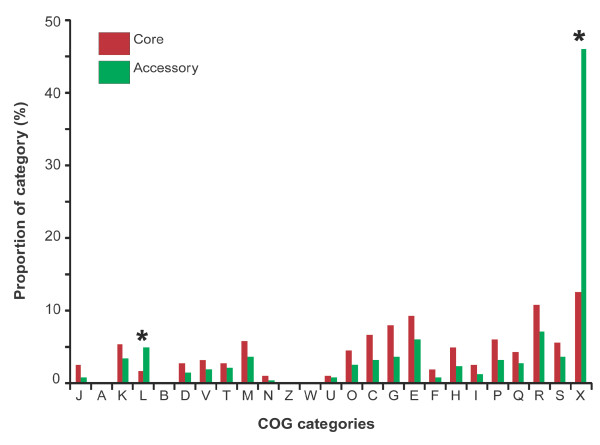
**Distribution of orthologs for each COG category in the core and the accessory genome; asterisks indicate those categories that are significantly different**. See Table S2 for COG codes.

In order to define possible differences in functions encoded by the core and/or the accessory genomes and by the different strains, each protein was assigned to a COG category (Additional file 2) and the abundance of each COG category was plotted (Figure 2, Additional file 3). Statistically significant differences between core and accessory genome were found only for COG category L (DNA replication, recombination and repair) and for proteins with no assigned COG (X): in these two categories, the accessory genome is enriched, especially in the category X. Similar enrichment in CDSs with no assigned function has been previously reported in the accessory genome of other organisms [21] as well as in the *S. meliloti *plasmid pSmeSM11a [22]. For other COG categories, no statistically significant difference was found, though a higher representation of all assigned functions was found in the core. Finally no significant difference of COG categories between the three strains was found (Additional file 3).

### Structural genomics

**Figure 3:**
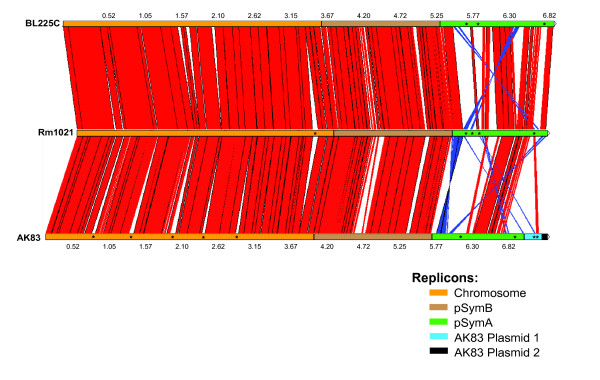
**Structure of *S. meliloti *genomes: genomic alignment of strain BL225C (top), Rm1021 (middle) and AK83 (bottom) with each replicon highlighted by different colours**. The size is expressed in Mbp. Red connections indicate syntenic regions, blue ones inversions, asterisks indicate the regions containing transposases as discussed in the text.

While BL225C contains three replicons as Rm1021, AK83 is composed by five circular replicons, corresponding to the chromosome, pSymA and pSymB, which are also present in the genome of Rm1021 and BL225C and two new small replicons 1 and 2, respectively 0.26 Mbp and 0.07 Mbp in size as schematized in Figure 3. The genomic structures of the fully assembled complete genomes were compared with a full-scale genomic alignment (see materials and methods). The chromosome and the pSymB chromid are characterized by a high resistance to genome rearrangements, with an almost perfect shared synteny, with only few insertions in the chromosome of strain AK83 and few rearranged regions of the chromid pSymB. The other replicons showed indeed lower degrees of synteny: in particular Plasmid2 of strain AK83 had no region of similarity with the other replicons of strain Rm1021 and BL225C (and with other plasmids available in the NCBI database). Concerning the symbiosis-required megaplasmid pSymA, a very low degree of synteny was observed indicating an increased rate of rearrangements for pSymA, and indeed evidences for rearrangements were noticed, since at least three fragments of Plasmid1 showed an high degree of similarity with the Rm1021 pSymA (ca. 47 kbp), while just one fragment was found to be highly similar to the pSymA of BL225C (ca. 27 kbp). These data suggest that AK83 Plasmid1 may be derived from or represent an evolutionary step toward the megaplasmid pSymA. Interestingly, a fragment 8 kbp long of Plasmid1 showed similarity with another symbiotic-related plasmid (*Sinorhizobium fredii *NGR234 plasmid pNGR234b). On the other hand, megaplasmid pSymA of strain BL225C compared to Rm1021 showed a higher number of syntenic regions and a lower number of inversions than AK83 (6 regions over 27 for the former and 11 regions over 24 for the latter). The genomic structure of the newly sequenced *S. meliloti *strain SM11 was also analyzed (data not shown); as expected SM11 pSmeSM11c (replicon carrying symbiotic functions, analogous to Rm1021 pSymA) is the most diverse replicon in comparison with replicons of strains AK83 and BL225C. No significant homologies were found between the small plasmids of strain AK83 (Plasmid 1 and Plasmid 2) and strain SM11 genome, as well as no significant hits between strain SM11 small plasmids (pSmeSM11a and pSmeSM11b) and AK83 and BL225C genomes. However, the presence on strain SM11 pSmeSM11a plasmid of two regions of 15 and 10 kbp syntenic to megaplsmid pSymA of strain Rm1021 was confirmed [22].

The location of transposases and insertion sequences was analyzed. Indeed in most of the cases, transposase encoding genes were enriched in regions carrying the traces of genomic rearrangements (with a frequency of 1.53, 1.32 and 0.59 IS/10 kb in RM1021, AK83 and BL225 respectively), than in highly syntenic regions where the corresponding frequencies are 0.19, 0.11 and 0.06 IS/10 kb in the three genomes. As reported in Figure 3, 17 transposases were found in a 80 kbp insertion of the chromosome of strain Rm1021 (3'357'000 to 3'440'000), as well as in the biggest five non-syntenic regions of the AK83 chromosome. In the pSymA megaplasmids, 13 transposases were found in the non-syntenic region of strain Rm1021 (200'000 to 390'000), 14 and 15 transposases were found in strain AK83 in two non-syntenyc regions (300'000 to 457'000 and 1'102'000 to 1'198'000) and in strain BL225C 17 and 2 transposases were found in two non-syntenyc regions (630'000 to 934'000 and 144'000 to 1'482'000). Finally, 4 transposases were found flanking the two regions of strain AK83 Plasmid1 that are also present on megaplasmid pSymA in strain Rm1021.

### Accessory genome and symbiosis-related functions

**Figure 4:**
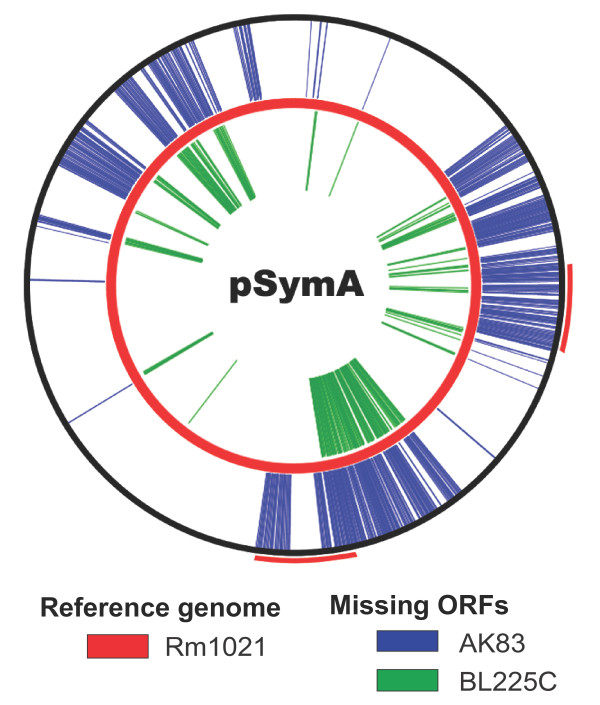
**Regions of Rm1021 pSymA megaplasmid (red circle) and regions absent in the genomes of strains AK83 (blue bars) and BL225C (green bars)**. The micro-aerophilic regions [24] are indicated with red external arches.

Looking at the genetic content of the symbiotic megaplasmid pSymA of Rm1021 in comparison with AK83 and BL225C, previous findings using CGH were confirmed [6,23]: in fact, many genes harbored by Rm1021 pSymA were found missing in the genomes of the two other strains (Figure 4). Moreover, two regions of Rm1021 pSymA (200'000 to 390'000 and 679'000 to 708'000), predicted to be a large part of the so-called microaerophilic gene set [24], were not present in strain AK83 and to a lesser extent also in strain BL225C.

We then focused on genes involved in some aspects of the symbiotic process, that we identified using a data mining strategy combining different sources of information, using the following approach: for each orthologous group having a predicted link to a NodMutDB and/or Rhizobase member (see materials and methods) the related literature was retrieved and analyzed to speculate its actual role in symbiosis. This approach was combined with other annotation sources (such as KEGG and Interpro) in a dedicated data mining procedure (Additional file 4); together with this approach, also those proteins with names containing symbiosis-related terms (e.g. fix, nif, nod) were retrieved. In particular we were interested in genes that are differentially present in the three strains and that could be related to the different symbiotic phenotypes of these strains.

By using the strategy described above, we identified, among the accessory genes, those that have been implicated in the symbiotic process in rhizobia through experimental work. Within a total number of 290 orthologous groups retrieved, 61 of them were found to belong to the accessory genome (21%) (Additional file 5).

These 61 accessory genes were divided in 36 entries (since 32 of them were organized in 7 operons): 22 of which were present in BL225C, 21 in Rm1021 and 12 in AK83 genomes (Table 2). Three main classes were identified, one comprising genes involved in microaerophilic growth during bacteroid development and putatively under the control of the transcriptional regulator FixK [2], the second comprising other genes either directly or indirectly related to symbiosis (e.g. affecting host range, nodule competitiveness, nodule number, nitrogen metabolism, etc.) and the latter comprising those proteins with limited information about their function, although probably involved in the symbiotic process. As mentioned before, genes belonging to the first class (microaerophilic gene set), were absent in the accessory genome of strain AK83 and present in both Rm1021 and BL225C strains (with the exception of *cycB2*, present in Rm1021 only). These genes are mainly clustered in three parts of the pSymA replicon of Rm1021, two of which known to be induced in microaerophilic conditions [24] (Figure 4). They include: a *fixK*-like gene encoding for a transcriptional regulator, the third copy of the operon *fixNOQP *encoding for an electron transport chain with high affinity to oxygen and a series of operons related to nitrogen metabolism: *nor *(nitric oxide reduction), *nir *(nitrite reduction), *nos *(nitrous oxide reduction), *nnr *(regulation of nir and nor operons), *nrt *(nitrate transport), plus two genes also related to the microaerophilic environment, *cycB2 *and *hemN*. Interestingly, we also identified different copy numbers of *fixK *genes in the three genomes: the actual regulator (belonging to the orthologous group N.280) was present in the core genome, FixK2 (orthologous group N.7042) was present only in Rm1021, and a FixK-like copy (orthologous group N.5488) was found in Rm1021 and BL225C genomes, as a part of the microaerophilic gene set; finally a FixK-like weak homolog was found in strains AK83 and BL225C (orthologous group N.5635). Other genes, present only in the accessory genome of Rm1021 and BL225C genomes, include a gene for a short chain dehydrogenase (SDR) whose mutation leads to the formation of white and elongated nodules [25] and the gene cluster for rhizobactin biosynthesis which, though not directly affecting nitrogen fixation [26], may have long-term effect on plant growth [27]. All together these two groups of accessory genes absent in AK83 genome may help explain for the reduced efficiency of plant growth promotion by this strain. Interestingly, only one symbiotic gene is absent just in strain BL225C; the first copy of the nodQ gene, the mutation of which leads to a slightly delayed nodulation [28-31]; since strain BL225C does not exhibit such a phenotype, it can be argued that probably this strain can overcome this gene loss, although the mechanism is still unclear. Among the remaining genes, 4 are exclusively present in strain AK83 including a nickel permease/hydrogenase *hup*E putatively involved in recycling the hydrogen developed during nitrogen fixation [32], a cadherin-like gene which may have auxiliary roles via Type I secretion system in cellular aggregation or attachment to roots [33], a CTP:phosphocholine cytidylyltransferase, involved in the phosphatididylcholine metabolism whose presence in the bacterial membrane is also important for the adhesion to eukaryotic cells [34] and the nodM gene, whose sequence is homologous but not orthologous to the same gene in the other two strains; since this gene plays a crucial role in the early steps of the rhizobial invasion of the host plant, this difference could have an impact on the symbiotic process. Two symbiotic genes are present in BL225C only, namely a phosphatidylcholine synthase distant homolog and the putative two-component response regulator gene *nws*B, which is related to strain competition for nodulation in *B. japonicum *[35]. Six genes are present in Rm1021 only: *cgmB*, the second and the third copies of *fixT*, the second copy of *nodP*, the second copy of *fixK *and the *expR *fragment; this means that *exp*R is disrupted in Rm1021 and therefore it doesn't give any functional products. The remaining genes include a homolog of the 5-aminolevulinate synthase (*hemA*) involved in the biosynthesis of porphyrins and putatively involved in the release of the rhizobial cells from the infection threads (absent in Rm1021) [36], one gene encoding a cytochrome P450 oxidase (CP450, absent in Rm1021), known to be expressed in bacteroids in other rhizobial species [37], the *acd*S gene encoding ACC deaminase (absent in Rm1021), which play a role in competition for nodulation [38], and a putative fucose isomerase (absent in AK83), which may add modifications to the Nod factor of strain Rm1021 and BL225C, thus potentially altering the communication with the host plant [39].

**Table 2:** Relevant genes of the accessory genome related to symbiotic interaction

Orthologous group(s)*	Gene or Protein Name	Strain(s)	Copies	Phenotype***	Species****	NodMutID
**Microaerophilic gene set**						
5488	*fixK-like*	Rm1021/BL225C	4	Nod+Fix-	*B. japonicum *USDA110	924-5
5305, 5324, 5377, 5379	*fixNOQP_3_*	Rm1021/BL225C	3	Nod+Fix-	*B. japonicum *USDA110	933-950
5327, 5300, 5361, 5326, 5378	*norBCDEQ*	Rm1021/BL225C	1	Nod+-	*B. japonicum *USDA110	921
5432, 5427, 5508, 5586, 5502, 5567, 5554	*nosRZDFYLX*	Rm1021/BL225C	1	Nod+Fix+	*B. japonicum *USDA110	1075
5498, 5537	*nirKV*	Rm1021/BL225C	1**	Nod+-	*B. japonicum *USDA110	922
5298, 5394, 5563	*nnrRSU*	Rm1021/BL225C	1**	N_2_metabolism	*S.meliloti *JJ1c10	---
5441, 5485, 5583	*nrtABC*	Rm1021/BL225C	1**	N_2_metabolism	*S.meliloti *Rm1021	---
7166	*cycB_2_*	Rm1021	2	Not known	*S. meliloti *Rm1021	---
5391	*hemN*	Rm1021/BL225C	1	Not known	*S. meliloti *JJ1c10	---
**Others**						
5422	*Symbiosis-related SDR*	Rm1021/BL225C	1	Nod+Fix+-	*S. meliloti *Rm1021	1310
6532	*nwsB*	BL225C	1	Nod+-	*B. japonicum USDA110*	911, 1015, 1019
5532, 5338, 5424, 548, 5381, 5346, 5437, 5522	*rhbABCDE, rhtAX, rhrA*	Rm1021/BL225C	1**	Nod+Fix+-	*S. meliloti *Rm1021	---
5832	*acdS*	AK83/BL225C	1	Nod+-	Several rhizobial species	---
5635	*fixKweakhomolog*	AK83/BL225C	4	Nod+Fix-	*B.japonicum *USDA110	924-5
7649	*nodQ_1_*	Rm1021/AK83	2	Nod+-	*S. meliloti *Rm1021	104, 119, 134-7, 618, 629
6799	*fixT_3_*	Rm1021	7	Not known	*S. meliloti *Rm1021	---
6905	*nodP_2_*	Rm1021	2	Host	*S. meliloti *Rm1021	---
7041	*fixT_2_*	Rm1021	7	Nod+Fix+	*S. meliloti *Rm1021	685
7042	*fixK_2_*	Rm1021	4	Nod+Fix-	*S. meliloti *Rm1021	489
5593	*C P450*	AK83/BL225C	3	Nod+Fix+	*B. japonicum *USDA110*, Rhizobium sp*. BR816	---
7427	*hupE*	AK83	1	Nod+Fix+	*A. caulinodans *ORS571	---
5770	*hemA *homolog	AK83/BL225C	1	Nod+Fix+-	*B. japonicum *USDA110	---
6640	*Pcs *distant homolog	BL225C	2	Host	Several bacterial species	---
7666	CTP:phosphocholinecytidylyltransferase	AK83	1	Host	Several bacterial species	---
7766	Cadherin-likeprotein	AK83	1	Host	*R. leguminosarum *bv.*viciae*	---
6766	*cgmB*	Rm1021	1	Host	*S. meliloti *Rm1021	---
6835	*expR *(fragment)	Rm1021	1	Not known	*S. meliloti *Rm1021	---
5551	Sugar isomerase	Rm1021/BL225C	1	Host	*S. meliloti *Rm1021	---
3183	*nodM *(AK83)	AK83	2	Host	*S. meliloti *Rm1021	---
**Not characterized**						
6353	*napC/nirT-like*	BL225C	1	N_2_metabolism	Several rhizobial species	---
8184	*glnA-like*	AK83	9	N_2_metabolism	Several rhizobial species	---
5573	*fixS2*	Rm1021/BL225C	2	Not known	*S. meliloti *Rm1021	---
5936	*fixO-like*	AK83/BL225C	2	Not known	Several rhizobial species	---
5950	*fixT1-like*	AK83/BL225C	7	Not known	Several rhizobial species	---
6498	*fixT-like*	BL225C	7	Not known	Several rhizobial species	---
7148	*fixL-related*	Rm1021	2	Not known	*S. meliloti *Rm1021	---

See text and Figure S1 for details of the searching procedure.

* See Table S1 for the accession numbers of the single proteins belonging to each group

** One or more genes are present in more than one copy

*** Host: recognition, communication and invasion of a host plant; Nod: nodulation phenotype; Fix: nitrogen fixation and plant growth promotion phenotype; + positive phenotype; +- slightly reduced phenotype; - absent phenotype

**** Organism in which the function of a specific protein or operon was elucidated

In conclusion, it can be noted that 48 orthologous groups are missing in the symbiosis defective AK83, which is, in fact, a large proportion of the accessory genes related to symbiosis (79%).

### The symbiosis-related panregulon

**Figure 5:**
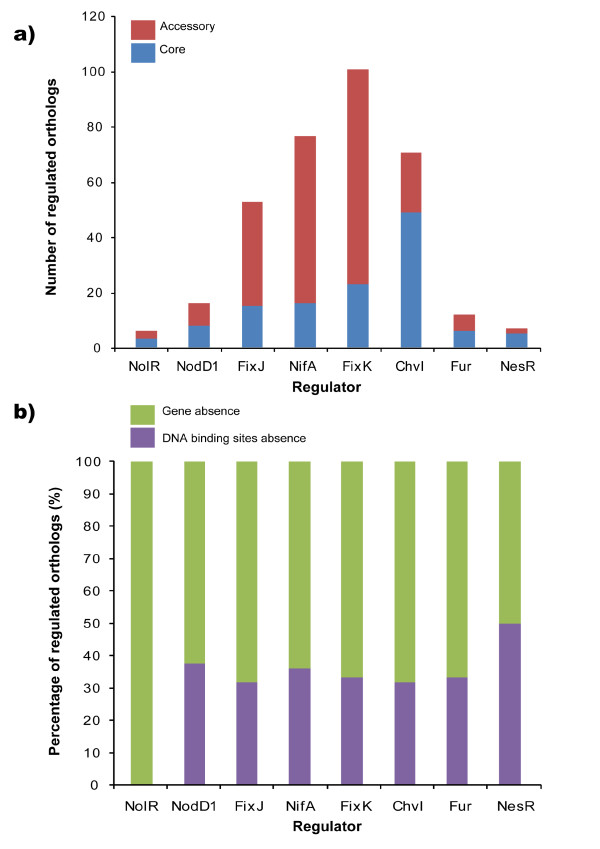
**General features of the panregulons for selected symbiosis-related transcriptional regulators; a) the overall number of putative target genes and their core and accessory fractions are indicated for each regulator; b) the fractions of regulons that are accessory due to the absence of the genes or the DNA-binding sites**.

To further investigate the genomic differences that may be related to the variable symbiotic phenotypes of AK83, BL225C and Rm1021 strains, the predicted regulons of a series of symbiotic transcription factors were analyzed. A set of eight transcriptional regulators related to symbiotic interaction was chosen, based on the knowledge of their DNA binding sites (Additional file 6) and on regulon information on closely related rhizobial species that may share the same binding site with *S. meliloti*. The set included transcriptional regulators involved in root exudates perception and early nodulation steps (NodD, NolR), microaerophilic adaptation (FixK), nitrogenase synthesis (FixJ, NifA), iron uptake (Fur), EPS biosynthesis (ChvI) and plant invasion competition (NesR) (Table 3). It should be noticed that the FixK regulated genes only partially overlap with those found to be expressed under microaerophilic conditions [24], which may be under control of other regulators. For each transcriptional regulator, genes putatively regulated and present in the genomes of strains Rm1021, AK83 and BL225C were sorted out by HMM scanning and the core (conserved in all strains) and the accessory (variable among strains) putative regulons were defined (Figure 5a). We defined the *panregulon *as the totality of gene families controlled by a specified transcription factor in a certain number of genomes, in analogy to the term pangenome [12], and which is formed by the core and the accessory regulons. The putative panregulon varies in sizes from 101 (FixK) to 6 (NolR) orthologous groups (i.e. genes); since some of the regulated targets could be part of an operon or be additional regulators, the actual size of the predicted panregulon is definitely under-estimated. NolR and NesR show a very little panregulon (with a core regulon of 3 and 5 orthologs, respectively); the accessory regulons of the other transcriptional regulators are very large and variable, accounting for 31-79% (average 55%) of all panregulons. The occurrence of wide accessory regulons could be due to the absence in one or two strains of the target genes or to the absence of the regulatory upstream sequences when the genes are present. The absence of target genes is the most frequent case ranging from 50% (NesR) to 100% (NolR) of the targeted genes, with an average value of 69% (Figure 5b). On the contrary the variability of DNA binding sites upstream CDSs (genes were still present in a given genome but were not putatively regulated by that transcription factor) is less frequent with an average value of 31%. The list of all genes sorted out as putatively regulated by the selected transcriptional regulators is reported in Additional file 6. The composition of the accessory regulons in terms of un-annotated targets was calculated counting the number of CDS with no COG classification (Additional file 7) resulting in an average percentage of 49%, while for the core regulons the percentages of un-annotated targets is lower (21%).

**Table 3:** Selected transcriptional regulators related to symbiosis with known binding site in *S. meliloti *(see Table S5 for consensus sequences)

			Genes regulated		
***Transcription factor***	***Symbiotic process***	***Reference***	***Rm1021***	***AK83***	***BL225C***
NodD1	Flavonoid perception	[40,68]	10	13	12
ChvI	EPS biosynthesis	[45]	54	65	52
FixK	Microaerophilic adaptation	[41]	54	61	54
FixJ	Nitrogenase synthesis and functioning via nifA	[69]	26	31	35
NifA	Nitrogenase biosynthesis	[41]	35	48	42
Fur	Iron uptake	[43]	9	8	9
NolR*	Optimization of nodulation, bacterial growth on solid medium, survival under stress conditions, and conjugative transfer of plasmids	[70]	3	6	3
NesR	Competition for plant nodulation	[44]	6	5	7

* gene disrupted by a frameshift mutation in Rm1021 [46]

**Figure 6:**
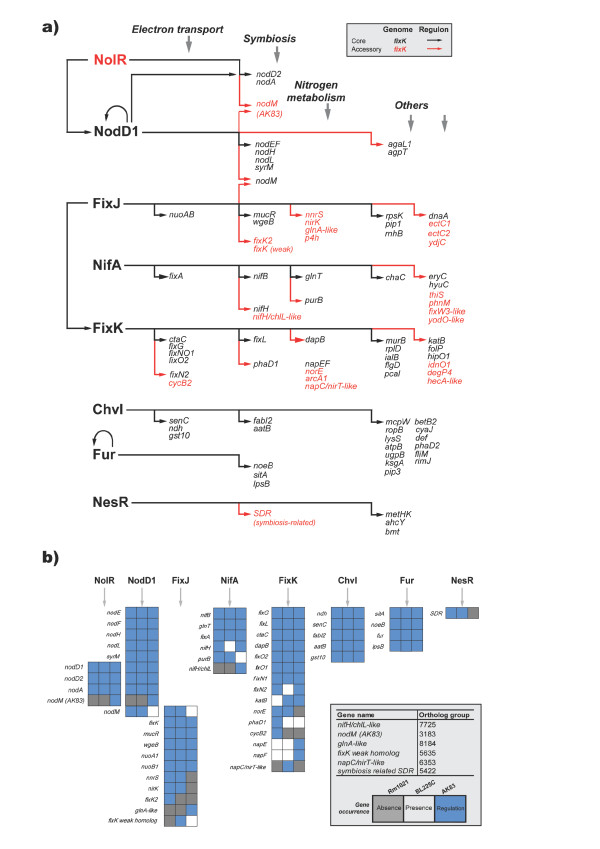
**Schematic diagram of the predicted regulons in all strains**. a) Putatively regulated genes have been vertically arranged in relation to their involvement in electron transport, symbiosis and nitrogen metabolism and other functions, while the genes without enough functional information are not reported in the diagram (see Table S3 for the complete list). Arrows indicate the presence of a predicted DNA-binding site upstream the indicated gene, with no inference about the role in the regulation of gene expression. Black gene names and arrows belong to core genome/regulons; red gene names and arrows belong to accessory genome/regulons. b) Details of the regulation of symbiosis-related genes among the three strains analyzed, Rm1021 (left), BL225C (middle) or AK83 (right); the color of the cell represents the absence of the gene (grey) or the presence of the gene (white, non-regulated or blue, regulated); only the scores above threshold are reported.

The genes of the predicted regulons were then divided into 5 functional groups: electron transport, symbiosis, nitrogen metabolism, others or un-annotated. In Figure 6a the eight regulons are showed; in many cases putatively regulated genes could be matched with experimental data: for NodD1 the experimental regulation of *nodL, nodF, nodA, nodM *and *syrM *[40] was confirmed by the analysis; for FixK the regulation of *fixNOQP_1_, fixNOQP_2_, fixGIS, arcABC, napEFDABC, norBCE, cycB2 *and *degP4 *was confirmed [41]; for NifA the regulation of *nifHDKEX, fixABCX *operons and the *nifB *genes [41] were also confirmed. Experimental confirmation of regulatory predictions by NolR was also found for *nodA, nodD1, nodD2 and nodM *[42], by Fur for *sitA *[43], by NesR for the *metHK *operon and the *ahcY *gene [44] and by ChvI for *ropB *[45]. Interestingly, even if the *nolR *gene is disrupted by a single base insertion in strain Rm1021 [46], the NolR binding sites in front of *nodD1, nodD2 *and *nodA *are maintained, suggesting that the inactivation may be relatively recent. Co-regulation by different factors was observed on several genes; in particular 7 genes were putatively regulated by more than one regulator (*nodD1, nodD2, nodA, nodM*, the AK83 copy of *nodM *and two other uncharacterized proteins) and a co-regulation by 4 out of the 8 selected regulators was also found (NolR on NodD1 and FixJ on FixK), indicating that some of the symbiotic regulators are linked together in a network that ensures the coordination of the expression of the genes required during infection and nitrogen fixation. The FixK regulator is predicted to control the highest number of genes involved in the symbiotic process, as well as those involved in nitrogen metabolism and electron transport needed in the microaerophilic environment of the bacteroid [24].

Concerning the variability in the predicted regulons (Figure 6b), it is evident that AK83, shows some differences in the regulatory networks: in AK83 *purB *is apparently not controlled by NifA; *nodM *is regulated by NolR only in strain AK83; in AK83 FixK controls *napE *and *napF*. It should be noted that these differences were not observable from patterns of gene presence/absence, illustrating the added value of regulon prediction in comparative genomics.

## Conclusions

The symbiosis between the nitrogen-fixing bacterium *S. meliloti *and the leguminous host plant *Medicago *is a case of a complex multigenic phenotype and one of the most deeply studied model systems [47]. In order to elucidate the genomic bases of the significant variability exhibited by environmental strains of the symbiotic phenotype, we sequenced the genomes of two strains of *S. meliloti*, AK83 and BL225C. These strains have different effects on plant growth, also in comparison with the reference strain Rm1021 [8].

We defined the core and the accessory genome of these three genomes as an approximation of the species pangenome, identifying a large set of genes, about 35% of the total number of genes annotated, belonging only to one or two of the strains analyzed; this proportion is similar to the pangenomic content of *Escherichia coli*, with ca. 42% of the genes belonging to the accessory genome, while in other species, such as *Bacillus anthracis *and *Streptococcus pneumoniae*, the size of the accessory genome is larger, 60% and 77% respectively. The *S. meliloti *pangenome elucidated in this work using three strains can be considered a good approximation of the species symbiotic pangenome, considering that the core genome size wasn't strongly affected by the addition of strain SM11.

The considerable number of accessory genes supports the vast phenotypic diversity of these strains and of the species [8].

Therefore we focused on genes, present in accessory genome, which were linked with the symbiotic process, using all literature and database data available. The approach, developed to aim at that purpose as depicted in Additional file 4, applied to the repertoire of symbiotic genes, had the advantage to speed up the data mining step, since any source of information was in the same database, allowing us to combine the results of various analyses. It should be emphasized that the procedure can be extended to any interesting phenotype for which genomic molecular information (genes) are available.

Symbiosis related genes have previously been shown to be highly variable among rhizobial species [48] To address the presence of an intra-specific variability in *S. meliloti*, a list of variable genes linked to symbiosis was compiled and analyzed trying to highlight the putative connection with the symbiotic phenotype of the three strains (AK83, BL225C, Rm1021). Surprisingly, the symbiotic accessory genome was found to be highly variable, including about 21% of all the symbiotic orthologs considered. We can then expect that different symbiotic phenotypes shown by *S. meliloti *strains may be indeed due to such high variability in the symbiotic accessory genome.

The most notable feature found was a large variability in the so-called "microaerophilic" gene set [24], which includes the transcriptional regulator annotated as FixK-like, a third copy of electron transport chain (*fixNOQP*) and several genes related to nitrogen metabolism (*nos, nor, nir, nnr *and *nrt*). These results confirm previous data obtained by CGH [6] and by phenotypic microarray on different metabolic activity of these strains in different nitrogen sources [8]. These findings, together with the lack of a symbiosis-related short chain dehydrogenase and the entire rhizobactin operon, may contribute to link the reduced plant height phenotype with the genomic structure of strain AK83. The inefficiency of symbiotic phenotype of strain AK83 was also confirmed by the observation of a relatively large number of immature nodules produced by AK83 on *M. truncatula *[8] and alfalfa (unpublished results). Consequently, the content of the accessory genome in the different strains can explain the differences in the symbiotic phenotype.

Even if the extent of the accessory genome by itself could account for the phenotypic differences between AK83 and BL225C/Rm1021, a comparable variability at regulatory level was also found. A set of regulons, defined by previous experimental work and known to be involved in the symbiotic process (the "symbiotic panregulon"), was investigated searching for the core regulon (putative regulatory interactions present in all the strains) and accessory regulon (regulatory interactions present in one or two strains). Again, a surprisingly large accessory regulon was found for most of the selected transcriptional regulators, either because of the absence of the target gene or because of the absence of the predicted regulator binding site. This result suggests that, other than gene content variation, regulons polymorphism could be a key determinant in the variability of symbiotic performances among strains.

The inclusion of regulatory networks in comparative genomic studies could represent a powerful extension of the analysis that can uncover the evolutionary events otherwise undetectable by gene presence comparison. The assumption behind this approach is that genetic modifications can occur in the structural gene and in the *cis *regulatory sequences leading to the same effect of the inactivation of the gene function. In the case of the symbiotic regulons of *S. meliloti*, we found that about 31% of the putatively missing connections between regulator and regulated genes are due the loss of DNA binding sites, the relative genes being still present in the genome. It can be conjectured that the presence of genes, which have lost (or not still acquired) the binding sites, may reflect a relatively recent evolutionary divergence, such as is expected among strains of the same species and confirmed in the case of *nolR*, whose inactivation in the laboratory strain Rm1021 probably happened recently, since even its DNA-binding sites are conserved.

In conclusion, we reported here a genomic analysis of the symbiotic variability at the intra-specific level in the non pathogenic α-proteobacterium *S. meliloti*. The analysis revealed an accessory genome fraction and regulatory variability large enough to shed light on the symbiotic differences of the strains. Moreover, several variable genes related to symbiotic diversity were clearly identified and their occurrence and putative regulation in the core and accessory genome was investigated. Finally, the approach used here on symbiotic genes could possibly be applied to other diverse phenotypes. The methods and the database set-up in the present work can constitute a powerful framework for the addition of other sequenced strains enabling the refinement of the pangenome and panregulon shape, and predicting new candidate genes responsible for symbiotic variability.

## Methods

### Bacterial strains and culture conditions

BL225C, isolated in Italy in an alfalfa field and AK83, isolated in the Aral sea region, were deposited at the German Collection of Microorganisms and Cell Cultures (DSMZ) with accession codes DSM23914 for strain BL225C and DSM23913 for strain AK83. AK83 strain is also present, as original specimen after initial isolation, in the culture collection of All-Russia Institute of Agricultural Microbiology (RIAM, St. Petersburg, Russia). *S*trains were cultured on solid or liquid TY medium [49] with 0.2 g/liter CaCO_3 _at 30°C.

### Whole-genome shotgun sequencing and draft annotation

Total DNA was isolated from *S. meliloti *AK83 and BL225C cultures with a CTAB method according to the recommended protocols by JGI-DOE http://my.jgi.doe.gov/general/. Genome sequencing was performed at the Joint Genome Institute (JGI) (Walnut Creek, California, USA) using a combination of Illumina [50] and 454 technologies [51]. The 454 Titanium standard data and the 454 paired end data were assembled together with Newbler, version 2.3. The Newbler consensus sequences were computationally shredded into 2 kb overlapping fake reads (shreds). Illumina sequencing data was assembled with VELVET, version 0.7.63 [52], and the consensus sequences were computationally shredded into 1.5 kb overlapping fake reads (shreds). The 454 Newbler consensus shreds, the Illumina VELVET consensus shreds and the read pairs in the 454 paired end library were integrated using parallel phrap, version 4.24 (High Performance Software, LLC). The software Consed [53-55] was used in the following finishing process. Illumina data was used to correct potential base errors and increase consensus quality using the software Polisher developed at JGI (Alla Lapidus, unpublished). Possible mis-assemblies were corrected using gapResolution (Cliff Han, unpublished), Dupfinisher [56], or sequencing cloned bridging PCR fragments with subcloning. The gaps between contigs in the genomes of strain AK83 and BL225C were closed by editing in Consed, by PCR and by Bubble PCR (J-F Cheng, unpublished) primer walks. For strain AK83 a total of 968 additional reactions and 11 shatter libraries were necessary to close gaps and to raise the quality of the finished sequence; the final assembly is based on 279.6 Mb of 454 draft data which provides an average 31.3 × coverage of the genome and 426 Mb of Illumina draft data which provides an average 62 × coverage of the genome. For strain BL225C a total of 801 additional reactions were necessary to close gaps and to raise the quality of the finished sequence. The final assembly is based on 290.2 Mb of 454 draft data which provides an average 27× coverage of the genome and 308 Mb of Illumina draft data which provides an average 44× coverage of the genome. For both genomes the gene model prediction and draft annotation was generated using Prodigal [57]. Sequences and annotation can be accessed through the JGI web site at the addresses http://genome.jgi-psf.org/sinma/sinma.home.html for AK83 and http://genome.jgi-psf.org/sinmb/sinmb.home.html for BL225C; both strains are being submitted in GenBank with the following master records [Genbank: NZ_AEDG01000000, Genbank: NZ_AEDH01000000] for BL225C and AK83, respectively.

### Annotation

Annotation was performed again on the three genomes using Blast+ 2.2.23 [58] and InterproScan 4.6 [59]. A bidirectional best blast hit (BBH) approach was used to annotate all the predicted proteins in the three genomes using the following three databases: NCBI nr, downloaded on May 18, 2010, all the Rhizobase http://genome.kazusa.or.jp/rhizobase/ proteomes, downloaded on June 1^st^, 2010 and the KEGG database http://www.genome.jp/kegg/, downloaded on May 26, 2010; for the first two databases an E-value threshold of 1e-10 was applied, while for the KEGG database a threshold of 1e-50 was applied. For the domain scan using InterproScan the Interpro database release 27 was used. All the results were linked to literature using the Interpro database, the UNIPROT database http://www.uniprot.org/ release 2010_06, the KEGG database, the Rhizobase Bibliome and the nodMutDB [5]. The number of predicted transposases and IS was inferred with a keyword search in the annotated protein set, while the number of putative Type III and Type IV secretion systems-related proteins, two component systems and ABC transporters was inferred searching specific interpro domains in each strain proteome.

### Structural Genomics

Genomic alignment between the complete genomes was generated using the megablast algorithm [58] retaining only hits of more than 10 kbp; the starting point of the replicons was changed in order to generate a clearer syntenic map with the Artemis Comparison Tool (ACT) from the Artemis suite [60]. The similarity of the two smaller plasmids of strain AK83 with other known plasmids was inspected using the megablast algorithm on the entire nucleotide NCBI database and retaining only those hits bigger than 5 kbp. The structure of the reference genome was compared to the newly sequenced genomes using the nucleotide sequence of each protein with a nucleotide Megablast with a 50% identity threshold: results were visualized using DnaPlotter from the Artemis suite [60].

### Orthology

Since the actual magnitude of a pangenome is computable only by sequencing each strain of the desired species [61], here we will refer to the term pangenome not as the full gene complement of the *S. meliloti *species, but only to the observable one.

The three proteomes were clustered into orthologous groups using a BBH approach through InParanoid 4.0 [62] and MultiParanoid [63]. The BLOSUM 80 matrix was used during the InParanoid run, while the "unique" flag was applied in the MultiParanoid run; MultiParanoid could be reliably used since the three genomes are evolutionary closely related. The pangenome size of the species *Escherichia coli, Bacillus anthracis *and *Streptococcus pneumoniae *were determined picking three complete proteomes at random from the NCBI public database and applying the same approach as *S.meliloti*; ten repetitions with different genomes were performed to calculate the average pangenome size.

### Functional Enrichment

To elucidate if the accessory genome was enriched in a particular function, the proportions of the COG categories [64] in the core and accessory genome were compared; to give statistical significance to the difference an enrichment analysis was performed, in a similar way as in Brilli et al. [19]; one million random samplings were performed and the COG proportions of each sample was compared to a sample from the whole genome. P-values below 0.05 were considered significant.

### Promoter prediction

Promoter prediction was performed by taking the nucleotide sequences in region -600 + 100 around the predicted gene start of all protein coding sequences in the three genomes. HMMer 3.0 [65] was used to build the promoter box HMMs (hmmbuild program) and to scan the promoter regions (hmmsearch program). The input alignments that generated the HMMs were retrieved from MEME [66] scans on sequences derived from literature. HMM scan was performed by switching off all the heuristic filters, collecting all the hits and calculating the score mean and standard deviation; after verification of the normality of the score distribution using Past [67] only those hits having a score greater than 3 standard deviation above the mean value were retained. For the prediction of the two FixJ DNA binding motifs, the results obtained were merged together.

### Data storage and scripting

All the collect data from annotation, orthology and promoter prediction were stored in a MySQL relational database and linked together in a proteome-centric way. All the analysis were performed using ad-hoc Python scripts, taking advantage of the BioPython and SciPy packages.

## Abbreviations

CDS: coding sequence; NCBI: National Center for Biotechnology Information; ORF: open reading frame; rRNA: ribosomial RNA; tRNA: RNA transfer; COG: cluster of orthologous groups; GO: gene ontology; KEGG: Kyoto encyclopedia of genes and genomes; IS: insertion sequence; HMM: hidden Markov model; MEME: multiple em for motif elicitation

## Authors' contributions

MG performed the bioinformatic analyses on *S. meliloti *genomes and contributed in writing the manuscript. EGB, AM and MBa conceived the idea, contributed in writing the manuscript and data interpretation. EGB, FP, AF, SM and MBr supported in experimental and bioinformatic analyses. SL, AL, J-FC, LC, SP, ML, LH, TW, NM, NI, HD, DB, CD, RT, CH and HT managed the JGI CSP project DE-AC02-05CH11231 and performed genome sequencing, finishing and annotation analyses. All authors read and approved the final manuscript

## Supplementary Material

Additional file 1 **The list of core and accessory proteins found in S. meliloti genomes**. The ortholog group, the organism (strain), the protein ID and its genopmic location are reported. Click here for file

Additional file 2 **List of COG codes**. The list of COG codes as reported at the URL: http://www.ncbi.nlm.nih.gov/COG/old/palox.cgi?fun=all is shown. Click here for file

Additional file 3 **Abundance of each COG category in the different strains**. The number of proteins belonging to each COG category is shown for Rm1021, AK83, BL225C strains. Click here for file

Additional file 4 **The data mining procedure followed for finding gene involved in symbiosis**. For each orthologous group having a predicted link to a NodMutDB and/or Rhizobase member the related literature was retrieved and analyzed to speculate its actual role in symbiosis. This approach was also combined with other annotation sources (such as KEGG and Interpro). Click here for file

Additional file 5 **Symbiosis ortholog groups**. Genes known to be involved in the symbiotic process from literature, from nodMutDB and for orthology with members of rhizobase are reported Click here for file

Additional file 6 **Symbiosis-related transcription factors**. The eight transcriptional regulators retrieved with indicated the genes putatively regulated in the three genomes are reported Click here for file

Additional file 7 **Percentage of hypothetical CDSs with no COG classification in the core and accessory regulon of selected transcriptional regulators**. The eight transcriptional regulators retrieved with indicated the percentages of hypothetical CDSs with no COG classification in the core and accessory regulon. Click here for file
