# Supplementary material for: Exploring the symbiotic pangenome of the nitrogen-fixing bacterium Sinorhizobium meliloti
Source: BMC Genomics. 2011 May 12;12:235. doi: 10.1186/1471-2164-12-235 (PMC3164228; doi:10.1186/1471-2164-12-235)
Supplement: Additional file 2 — List of COG codes. The list of COG codes as reported at the URL: http://www.ncbi.nlm.nih.gov/COG/old/palox.cgi?fun=all is shown. [file 1471-2164-12-235-S2.DOCX]

**Table S2**. List of COG codes (from http://www.ncbi.nlm.nih.gov/COG/old/palox.cgi?fun=all)

| **Code** | **Description** |
| --- | --- |
|  |  |
|  |  |
|  |  |
| [J](http://www.ncbi.nlm.nih.gov/COG/old/palox.cgi?fun=J) | Translation, ribosomal structure and biogenesis |
| [K](http://www.ncbi.nlm.nih.gov/COG/old/palox.cgi?fun=K) | Transcription |
| [L](http://www.ncbi.nlm.nih.gov/COG/old/palox.cgi?fun=L) | DNA replication, recombination and repair |
| [D](http://www.ncbi.nlm.nih.gov/COG/old/palox.cgi?fun=D) | Cell division and chromosome partitioning |
| [O](http://www.ncbi.nlm.nih.gov/COG/old/palox.cgi?fun=O) | Posttranslational modification, protein turnover, chaperones |
| [M](http://www.ncbi.nlm.nih.gov/COG/old/palox.cgi?fun=M) | Cell envelope biogenesis, outer membrane |
| [N](http://www.ncbi.nlm.nih.gov/COG/old/palox.cgi?fun=N) | Cell motility and secretion |
| [P](http://www.ncbi.nlm.nih.gov/COG/old/palox.cgi?fun=P) | Inorganic ion transport and metabolism |
| [T](http://www.ncbi.nlm.nih.gov/COG/old/palox.cgi?fun=T) | Signal transduction mechanisms |
| [C](http://www.ncbi.nlm.nih.gov/COG/old/palox.cgi?fun=C) | Energy production and conversion |
| [G](http://www.ncbi.nlm.nih.gov/COG/old/palox.cgi?fun=G) | Carbohydrate transport and metabolism |
| [E](http://www.ncbi.nlm.nih.gov/COG/old/palox.cgi?fun=E) | Amino acid transport and metabolism |
| [F](http://www.ncbi.nlm.nih.gov/COG/old/palox.cgi?fun=F) | Nucleotide transport and metabolism |
| [H](http://www.ncbi.nlm.nih.gov/COG/old/palox.cgi?fun=H) | Coenzyme metabolism |
| [I](http://www.ncbi.nlm.nih.gov/COG/old/palox.cgi?fun=I) | Lipid metabolism |
| [Q](http://www.ncbi.nlm.nih.gov/COG/old/palox.cgi?fun=Q) | Secondary metabolites biosynthesis, transport and catabolism |
| [R](http://www.ncbi.nlm.nih.gov/COG/old/palox.cgi?fun=R) | General function prediction only |
| [S](http://www.ncbi.nlm.nih.gov/COG/old/palox.cgi?fun=S) | Function unknown |
| [X](http://www.ncbi.nlm.nih.gov/COG/old/palox.cgi?fun=S) | No COG category assigned |
