# Supplementary material for: Exploring the symbiotic pangenome of the nitrogen-fixing bacterium Sinorhizobium meliloti
Source: BMC Genomics. 2011 May 12;12:235. doi: 10.1186/1471-2164-12-235 (PMC3164228; doi:10.1186/1471-2164-12-235)
Supplement: Additional file 3 — Abundance of each COG category in the different strains. The number of proteins belonging to each COG category is shown for Rm1021, AK83, BL225C strains. [file 1471-2164-12-235-S3.DOCX]

**Table S3.** Abundance of each COG category in the different strains.

| **COG** | **Rm1021** | **AK83** | **BL225C** |
| --- | --- | --- | --- |
| **J** | 213 | 212 | 210 |
| **A** | 0 | 0 | 0 |
| **K** | 507 | 514 | 524 |
| **L** | 259 | 250 | 207 |
| **B** | 1 | 1 | 1 |
| **D** | 250 | 249 | 246 |
| **V** | 300 | 301 | 291 |
| **T** | 268 | 245 | 265 |
| **M** | 543 | 542 | 547 |
| **N** | 87 | 88 | 89 |
| **Z** | 0 | 0 | 0 |
| **W** | 1 | 1 | 2 |
| **U** | 82 | 92 | 89 |
| **O** | 419 | 428 | 420 |
| **C** | 620 | 595 | 621 |
| **G** | 753 | 722 | 758 |
| **E** | 903 | 895 | 904 |
| **F** | 163 | 159 | 162 |
| **H** | 453 | 444 | 454 |
| **I** | 232 | 227 | 235 |
| **P** | 575 | 552 | 556 |
| **Q** | 406 | 398 | 407 |
| **R** | 1044 | 1025 | 1052 |
| **S** | 529 | 513 | 533 |
| **X** | 1475 | 1881 | 1564 |
