# Supplementary figures and images for: Exploring the symbiotic pangenome of the nitrogen-fixing bacterium Sinorhizobium meliloti
Source: BMC Genomics. 2011 May 12;12:235. doi: 10.1186/1471-2164-12-235 (PMC3164228; doi:10.1186/1471-2164-12-235)

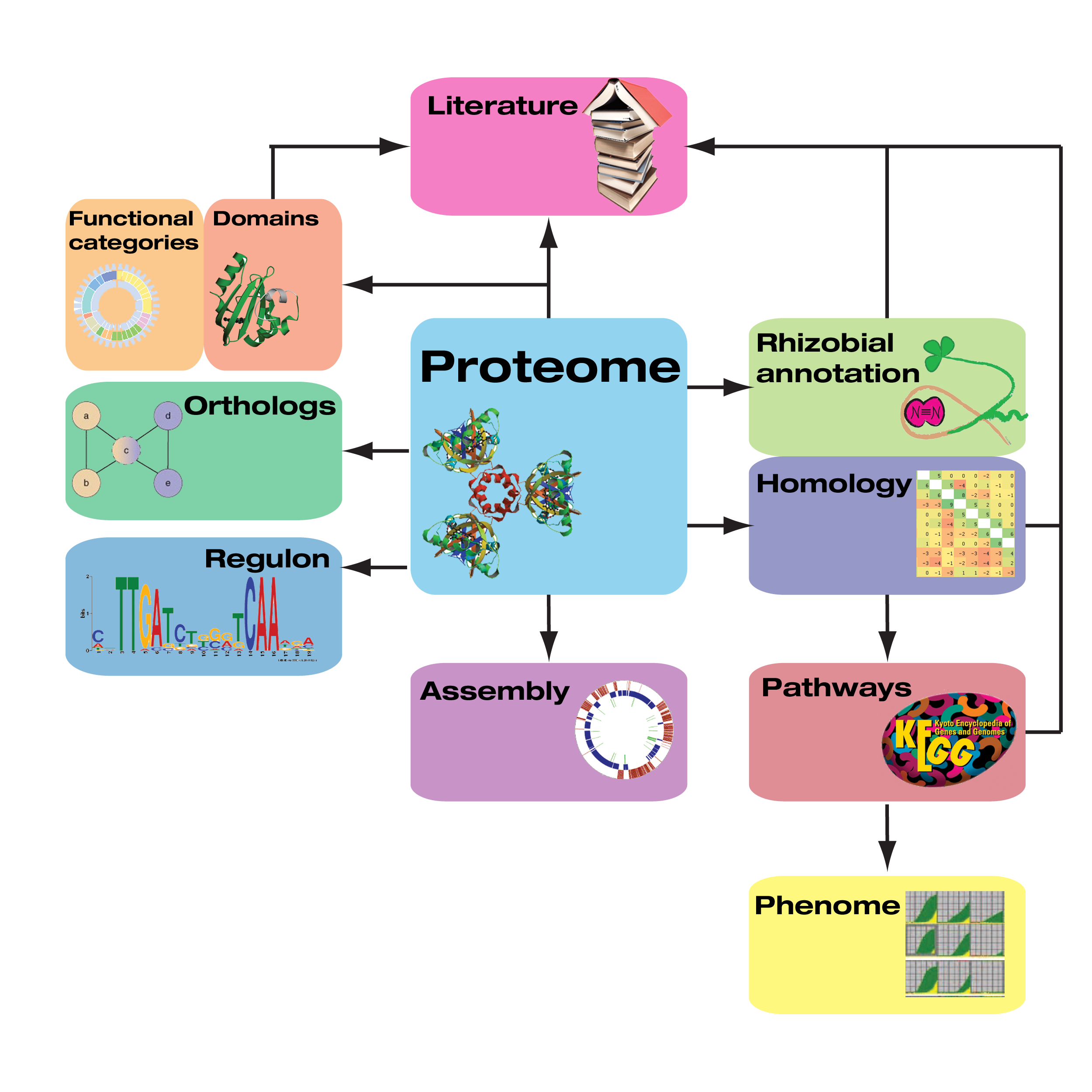

Supplement: Additional file 4 — The data mining procedure followed for finding gene involved in symbiosis. For each orthologous group having a predicted link to a NodMutDB and/or Rhizobase member the related literature was retrieved and analyzed to speculate its actual role in symbiosis. This approach was also combined with other annotation sources (such as KEGG and Interpro). [file 1471-2164-12-235-S4.PNG]
