# Supplementary material for: Exploring the symbiotic pangenome of the nitrogen-fixing bacterium Sinorhizobium meliloti
Source: BMC Genomics. 2011 May 12;12:235. doi: 10.1186/1471-2164-12-235 (PMC3164228; doi:10.1186/1471-2164-12-235)
Supplement: Additional file 5 — Symbiosis ortholog groups. Genes known to be involved in the symbiotic process from literature, from nodMutDB and for orthology with members of rhizobase are reported [file 1471-2164-12-235-S5.PDF]

Symbiosis ortholog groups

Genes known to be involved in the symbiotic process from literature, from nodMutDB and for orthology with members of rhizobase

Gene regulated
Gene present
Gene absent

Regulators

		Molecule			NolR			NodD1			FixJ			NifA			FixK			ChvI			Fur			NesR		
Ortholog	Gene name	Rm1021	AK83	BL225C	Rm1021	AK83	BL225C	Rm1021	AK83	BL225C	Rm1021	AK83	BL225C	Rm1021	AK83	BL225C	Rm1021	AK83	BL225C	Rm1021	AK83	BL225C	Rm1021	AK83	BL225C	Rm1021	AK83	BL225C
2248	nodD2	pSymA	pSymA	pSymA																								
4778	nodD1	pSymA	pSymA	pSymA																								
3975	nodA	pSymA	pSymA	pSymA																								
3183	nodM (AK83)		pSymA																									
1218	nodE	pSymA	pSymA	pSymA																								
1918	nodF	pSymA	pSymA	pSymA																								
2319	nodH	pSymA	pSymA	pSymA																								
840	nodL	pSymA	pSymA	pSymA																								
161	syrM	pSymA	pSymA	pSymA																								
6104	nodM	pSymA	Chr	pSymA																								
280	fixK	pSymA	pSymA	pSymA																								
4516	mucR	Chr	Chr	Chr																								
4028	wgeB	pSymB	pSymB	pSymB																								
2027	nuoA1	Chr	Chr	Chr																								
1269	nuoB1	Chr	Chr	Chr																								
5394	nnrS	pSymA		pSymA																								
5498	nirK	pSymA		pSymA																								
7042	fixK2	pSymA																										
8184	glnA-like		Plasmid1																									
5635	fixK weak homolog		pSymA	pSymA																								
5187	nifB	pSymA	pSymA	pSymA																								
5058	glnT	Chr	Chr	Chr																								
1855	fixA	pSymA	pSymA	pSymA																								
5235	nifH	pSymA	pSymA	pSymA																								
5042	purB	Chr	Chr	Chr																								
7725	nifH/chlL-like		Chr																									
2024	fixG	pSymA	pSymA	pSymA																								
3063	fixL	pSymA	pSymA	pSymA																								
1902	ctaC	Chr	Chr	Chr																								
3414	dapB	Chr	Chr	Chr																								
5939	fixO2	pSymA	pSymA	pSymA																								
4757	fixo1	pSymA	pSymA	pSymA																								
4714	fixn1	pSymA	pSymA	pSymA																								
402	fixN2	pSymA	pSymA	pSymA																								
374	katB	pSymA	pSymA	pSymA																								
5378	norE	pSymA		pSymA																								
3903	phaD1	Chr	Chr	Chr																								
7166	cycB2	pSymA																										
2292	napE	pSymA	pSymA	pSymA																								
4937	napF	pSymA	pSymA	pSymA																								
6353	napC/nirT-like			pSymA																								
5181	ndh	Chr	Chr	Chr																								
2956	senC	Chr	Chr	Chr																								
1031	fabI2	Chr	Chr	Chr																								
1479	aatB	Chr	Chr	Chr																								
5157	gst10	Chr	Chr	Chr																								
4278	sitA	Chr	Chr	Chr																								
187	noeB	pSymA	pSymA	pSymA																								
4320	fur	Chr	Chr	Chr																								
3250	lpsB	Chr	Chr	Chr																								
5422	symbiosis related SDR	pSymA		pSymA																								
5488	fixK	pSymA		pSymA																								
5305	fixO3	pSymA		pSymA																								
5324	fixN3	pSymA		pSymA																								
5377	fixQ3	pSymA		pSymA																								
5327	norD	pSymA		pSymA																								
5300	norQ	pSymA		pSymA																								
5361	norB	pSymA		pSymA																								
5326	norC	pSymA		pSymA																								
5508	nosD	pSymA		pSymA																								
5586	nosF	pSymA		pSymA																								
5502	nosY	pSymA		pSymA																								
5567	nosL	pSymA		pSymA																								
5554	nosX	pSymA		pSymA																								

5537	nirV	pSymA		pSymA
5298	nnrR	pSymA		pSymA
5441	nrtB	pSymA		pSymA
5485	nrtC	pSymA		pSymA
5583	nrtA	pSymA		pSymA
5391	hemN	pSymA		pSymA
5338	rhbA	pSymA		pSymA
5424	rhbB	pSymA		pSymA
5346	rhbE	pSymA		pSymA
5437	rhrA	pSymA		pSymA
5522	rhtA	pSymA		pSymA
5551	L-arabinose isomerase protein	pSymB		pSymB
5379	fixP3	pSymA		pSymA
5482	fixI2	pSymA		pSymA
5427	nosZ	pSymA		pSymA
5573	fixS2	pSymA		pSymA
5381	rhbD	pSymA		pSymA
5432	nosR	pSymA		pSymA
5563	nnrU	pSymA		pSymA
7649	nodQ1	pSymA	pSymA	
5832	acdS		pSymA	pSymA
5593	cytochrome P450		pSymA	pSymA
5770	hemA homolog		pSymA	pSymA
5936	fixO-like		pSymA	pSymA
5950	fixT1-like		pSymA	pSymA
6532	nwsB			pSymA
6640	pcs distant homolog			pSymA
6498	fixT-like			pSymA
6766	cgmB	Chr		
6835	expR (fragment)	Chr		
7148	FixL-related histidine kinase	pSymA		
6905	nodP2	pSymB		
7041	fixT2	pSymA		
6799	fixT3	Chr		
7427	hupE		Chr	
7666	CTP:phosphocholine cytidylyltransferase		Plasmid2	
7766	Cadherin-like protein		pSymB	
5037	etfB1	Chr	Chr	Chr
4534	hemB	Chr	Chr	Chr
2109	nifA	pSymA	pSymA	pSymA
2664	groEL	Chr-pSymA	Chr-pSymA	Chr-pSymA
1748	lsrB	Chr	Chr	Chr
5078	ptsN	Chr	Chr	Chr
2676	phaA1	Chr	Chr	Chr
3612	phaC1	Chr	Chr	Chr
2771	fixT1	pSymA	pSymA	pSymA
2531	exoD-like	pSymB	pSymB	pSymB
597	fixP1	pSymA	pSymA	pSymA
4848	fixS1	pSymA	pSymA	pSymA
4077	fixI1	pSymA	pSymA	pSymA
1631	fixJ	pSymA	pSymA	pSymA
4418	fixM	pSymA	pSymA	pSymA
2816	wgeA	pSymB	pSymB	pSymB
540	nifN	pSymA	pSymA	pSymA
548	carA	Chr	Chr	Chr
582	fixQ2	pSymA	pSymA	pSymA
1141	fixP2	pSymA	pSymA	pSymA
1237	fixU	pSymA	pSymA	pSymA
1245	fixX	pSymA	pSymA	pSymA
2909	fixC	pSymA	pSymA	pSymA
2726	fixB	pSymA	pSymA	pSymA
2937	fixH	pSymA	pSymA	pSymA
1764	fixQ1	pSymA	pSymA	pSymA
3950	nifU-like	Chr	Chr	Chr
685	nifX	pSymA	pSymA	pSymA
2463	nifS	Chr	Chr	Chr
2906	nifD	pSymA	pSymA	pSymA
236	nifK	pSymA	pSymA	pSymA
626	nifE	pSymA	pSymA	pSymA
1686	nifU-like	Chr	Chr	Chr
1069	nodD3	pSymA	pSymA	pSymA
5921	nodP1	pSymA	pSymB-pSy	pSymB
1273	nfeD	pSymB	pSymB	pSymB
1635	nodJ	pSymA	pSymA	pSymA

1711	nodI	pSymA	pSymA	pSymA
186	nodN	pSymA	pSymA	pSymA
2232	nodC	pSymA	pSymA	pSymA
2350	nodL-like	pSymB	pSymB	pSymB
2413	nodQ2	pSymB	pSymB	pSymB
4419	nodN2	Chr	Chr	Chr
4608	noeA	pSymA	pSymA	pSymA
4696	nodB	pSymA	pSymA	pSymA
4969	nodG	pSymA	pSymA	pSymA
269	nolG	pSymA	pSymA	pSymA
3884	nolF	pSymA	pSymA	pSymA
5240	chvG	Chr	Chr	Chr
53	chvI	Chr	Chr	Chr
2200	chvE	pSymB	pSymB	pSymB
46	cysG	Chr	Chr	Chr
71	thiO	pSymB	pSymB	pSymB
72	exoX	pSymB	pSymB	pSymB
84	rpoC	Chr	Chr	Chr
112	glnB	Chr	Chr	Chr
125	ndvB	Chr	Chr	Chr
146	leuB	Chr	Chr	Chr
6082	wgaA0	pSymB	pSymB	pSymB
318	exoH	pSymB	pSymB	pSymB
399	ilvD2	Chr	Chr	Chr
491	tpiA	Chr	Chr	Chr
527	purL	Chr	Chr	Chr
576	phoT	pSymB	pSymB	pSymB
615	leuA1	Chr	Chr	Chr
686	phoU	Chr	Chr	Chr
712	wgaE	pSymB	pSymB	pSymB
720	exoY	pSymB	pSymB	pSymB
725	gtrA	pSymA	pSymA	pSymA
741	wgeH	pSymB	pSymB	pSymB
768	rkpZ1	pSymB	pSymB	pSymB
810	rkpZ2	pSymB	pSymB	pSymB
823	msbB	Chr	Chr	Chr
839	queA	Chr	Chr	Chr
903	emmC	pSymB	pSymB	pSymB
921	sinI	Chr	Chr	Chr
942	phoE	pSymB	pSymB	pSymB
962	symbiosis related SDR	Chr	Chr	Chr
978	expR	Chr	Chr	Chr
1010	rpoB	Chr	Chr	Chr
1045	exoD	Chr	Chr	Chr
1311	wgcA	pSymB	pSymB	pSymB
1387	wgaB	pSymB	pSymB	pSymB
1410	fabI1	Chr	Chr	Chr
1416	exoB	pSymB	pSymB	pSymB
1421	bioS	Chr	Chr	Chr
1465	exoK	pSymB	pSymB	pSymB
1823	wgaA	pSymB	pSymB	pSymB
1472	metA	Chr	Chr	Chr
1541	exoA	pSymB	pSymB	pSymB
1552	psd	Chr	Chr	Chr
1561	wgeC	pSymB	pSymB	pSymB
1670	tkt2	Chr	Chr	Chr
1700	leuD	Chr	Chr	Chr
1730	emmB	pSymB	pSymB	pSymB
1758	exoF1	pSymB	pSymB	pSymB
1817	exoN	pSymB	pSymB	pSymB
1908	lpsE	Chr	Chr	Chr
1935	pckA	Chr	Chr	Chr
1944	exoZ	pSymB	pSymB	pSymB
1946	exoP	pSymB	pSymB	pSymB
1963	catC	pSymB	pSymB	pSymB
2238	relA	Chr	Chr	Chr
2259	cycH	Chr	Chr	Chr
2274	msbA2	pSymB	pSymB	pSymB
2311	feuP	Chr	Chr	Chr
2436	ntrR1	Chr	Chr	Chr
2449	lpsL	Chr	Chr	Chr
2479	thiD	pSymB	pSymB	pSymB
2498	pgm	Chr	Chr	Chr
2522	tolC	Chr	Chr	Chr

2545	exoT	pSymB	pSymB	pSymB
2551	exoO	pSymB	pSymB	pSymB
2557	glgA	Chr	Chr	Chr
2571	hemA	Chr	Chr	Chr
2575	pyrE	Chr	Chr	Chr
2609	symbiosis related SDR	pSymB	pSymB	pSymB
2612	glnD	Chr	Chr	Chr
2618	cbrA	Chr	Chr	Chr
2647	trpE	Chr	Chr	Chr
2688	rpoH1	Chr	Chr	Chr
2699	symbiosis related SDR	pSymB	pSymB	pSymB
2737	hprK	Chr	Chr	Chr
2823	dctD	pSymB	pSymB	pSymB
2837	rkpK	Chr	Chr	Chr
2862	dctA	pSymB	pSymB	pSymB
2870	mdh	Chr	Chr	Chr
2907	glgA	pSymB	pSymB	pSymB
2945	typA	Chr	Chr	Chr
2958	lpsS	Chr	Chr	Chr
2967	cycK	Chr	Chr	Chr
2987	exoR	Chr	Chr	Chr
3003	olsB	Chr	Chr	Chr
3036	gtrB	pSymA	pSymA	pSymA
3052	thiC	pSymB	pSymB	pSymB
3066	exoQ	pSymB	pSymB	pSymB
3074	katA	Chr	Chr	Chr
3094	tpiA	Chr	Chr	Chr
3107	symbiosis related SDR	pSymB	pSymB	pSymB
3115	olsA	Chr	Chr	Chr
3134	bluB	Chr	Chr	Chr
3159	exoL	pSymB	pSymB	pSymB
3337	ddhB	pSymB	pSymB	pSymB
3360	lon	Chr	Chr	Chr
3375	exoV	pSymB	pSymB	pSymB
3389	ilvC	Chr	Chr	Chr
3410	acpXL	Chr	Chr	Chr
3534	sodB	Chr	Chr	Chr
3621	exoU	pSymB	pSymB	pSymB
3623	pit	Chr	Chr	Chr
3660	isrA	Chr	Chr	Chr
3704	symbiosis related SDR	pSymB	pSymB	pSymB
3763	phbB	Chr	Chr	Chr
3815	cpdR1	Chr	Chr	Chr
3827	ndvA	Chr	Chr	Chr
3846	minE	pSymB	pSymB	pSymB
3918	aniA	Chr	Chr	Chr
3949	tme	Chr	Chr	Chr
3958	wgdB	pSymB	pSymB	pSymB
4047	rpoN	Chr	Chr	Chr
4064	ntrP	Chr	Chr	Chr
4153	lpsC	Chr	Chr	Chr
4157	ccmC	Chr	Chr	Chr
4232	degP1	Chr	Chr	Chr
4238	feuQ	Chr	Chr	Chr
4251	fdxN	pSymA	pSymA	pSymA
4293	wgdA	pSymB	pSymB	pSymB
4342	wgeG	pSymB	pSymB	pSymB
4355	leuC	Chr	Chr	Chr
4378	phoD	pSymB	pSymB	pSymB
4436	pth	Chr	Chr	Chr
4445	rpoH2	Chr	Chr	Chr
4450	emmA	pSymB	pSymB	pSymB
4479	phbC	Chr	Chr	Chr
4520	dctB	pSymB	pSymB	pSymB
4654	ilvI	Chr	Chr	Chr
4657	exoW	pSymB	pSymB	pSymB
4662	phoC	pSymB	pSymB	pSymB
4766	symbiosis related hypothetical protein	Chr	Chr	Chr
4780	wggR	pSymB	pSymB	pSymB
4807	flgH	Chr	Chr	Chr
4895	ppiB	Chr	Chr	Chr
5016	dme	Chr	Chr	Chr
5017	ntrC	Chr	Chr	Chr
5029	bacA	pSymB	pSymB	pSymB

5140	wgaH	pSymB	pSymB	pSymB									
5863	pcm	Chr	Chr	Chr									
5210	exoM	pSymB	pSymB	pSymB									
5247	greA	Chr	Chr	Chr									
