# Supplementary material for: Exploring the symbiotic pangenome of the nitrogen-fixing bacterium Sinorhizobium meliloti
Source: BMC Genomics. 2011 May 12;12:235. doi: 10.1186/1471-2164-12-235 (PMC3164228; doi:10.1186/1471-2164-12-235)
Supplement: Additional file 7 — Percentage of hypothetical CDSs with no COG classification in the core and accessory regulon of selected transcriptional regulators. The eight transcriptional regulators retrieved with indicated the percentages of hypothetical CDSs with no COG classification in the core and accessory regulon. [file 1471-2164-12-235-S7.DOC]

**Table S5**. Percentage of hypothetical CDSs with no COG classification in the core and accessory regulon of selected transcriptional regulators

|  | NolR | NodD1 | FixJ | NifA | FixK | ChvI | Fur | NesR | NolR |
| --- | --- | --- | --- | --- | --- | --- | --- | --- | --- |
| **% hyp. CDSs over core regulon** | 0.33 | 0.38 | 0.13 | 0.19 | 0.09 | 0.22 | 0.33 | 0 | 0.33 |
| **% hyp. CDSs over accessory regulon** | 0.66 | 0.38 | 0.45 | 0.59 | 0.53 | 0.5 | 0.83 | 0 | 0.67 |
